# Incidence of Ulnar Collateral Ligament Surgery and Revision in Baseball Players: A Systematic Review and Meta-Analysis

**DOI:** 10.3390/sports13090299

**Published:** 2025-09-01

**Authors:** Alberto Sáez, Gonzalo Mariscal, Carlos Barrios

**Affiliations:** Institute for Research on Musculoskeletal Disorders, School of Medicine, Valencia Catholic University, 46001 Valencia, Spain; alberto.saez@mail.ucv.es (A.S.); carlos.barrios@ucv.es (C.B.)

**Keywords:** baseball, elbow, ulnar collateral ligament, UCL injury, incidence, UCL reconstruction, Tommy John surgery, epidemiology, meta-analysis

## Abstract

Ulnar collateral ligament (UCL) injuries are a major concern in baseball because of repetitive valgus stress from overhead throwing, often leading to surgical intervention. In recent years, UCL reconstruction has become increasingly frequent. Although this procedure has a high success rate, revision surgery is often required. This systematic review and meta-analysis examined the incidence of UCL surgery and revision procedures in baseball players, explored potential risk factors, and identified areas for future research. A comprehensive search of PubMed, Google Scholar, CINAHL, Embase, and SportDiscus databases was conducted for studies published between 2014 and 2024. Studies reporting the incidence of primary and revision UCL reconstructions in baseball players were included. The quality of the included studies was assessed using the Methodological Index for Non-randomized Studies (MINORS). A meta-analysis using RevMan 5.4 software calculated the pooled proportions and 95% confidence intervals for UCL surgery and revision rates. Six studies met the inclusion criteria, including data from 27,366 baseball players. The pooled incidence of UCL surgery was 4.52% (95% CI: 4.20–4.84%), and the pooled incidence of revision surgery was 8.42% (95% CI: 5.49–11.35%). Statistical analyses showed high heterogeneity (I^2^ = 100%, *p* < 0.00001). Sensitivity analyses were performed to assess the robustness of the results, both in the incidence of surgery and in the revision surgery. UCL surgery is a common procedure in baseball, and a notable proportion of athletes require revision. Further research is necessary to identify the risk factors and develop preventive strategies to reduce UCL injury rates.

## 1. Introduction

Historically considered a career-terminating injury for elite pitchers, the prognosis of ulnar collateral ligament (UCL) insufficiency was revolutionized by Dr. Frank Jobe’s 1974 UCL reconstruction (UCLR) on Tommy John—a procedure now eponymously recognized as “Tommy John surgery” [1]. In recent decades, the incidence of UCL injuries necessitating surgical intervention has exhibited a marked upward trajectory. Between 2002 and 2011, the annual volume of UCL reconstruction (UCLR) surged by 193%, with population-adjusted rates tripling from 0.15 to 0.45 per 100,000 individuals [2]. Concurrently, the performance of concomitant ulnar nerve decompression or transposition procedures during UCLR increased by 400% during this period, reflecting evolving surgical practices (*p* < 0.001) [2]. National data confirm this trend beyond baseball. From 2007 to 2011, 790 UCLR procedures were recorded, predominantly in males (7.3:1) and most frequently in adolescents aged 15–19 years (79%), followed by those aged 20–24 years [3].

The extreme valgus stress of pitching renders UCL injuries highly prevalent in baseball. The economic impact is substantial—rehabilitation for Major League Baseball (MLB) pitchers is estimated at USD 395 million—with only 77% returning to play at the professional level [4]. While primary UCLR achieves favorable outcomes, revision surgery is required in 6.6% of cases [5]. Since the inaugural 1974 procedure, UCLR utilization has escalated precipitously among professional pitchers, reaching 33 primary reconstructions in 2012 [1]. Camp et al. (2018) conducted a comprehensive epidemiological assessment of 1.429 Major League Baseball/Minor League Baseball (MLB/MiLB) pitchers who underwent UCLR between 1974 and 2016, identifying 93.4% as primary procedures and 6.6% as revisions [6]. Notably, annual surgical rates have demonstrated consistent increases across all cohorts, including MLB, MiLB, and revision-specific procedures [6]. Position-specific analyses revealed a disproportionate injury burden among pitchers, with 16% requiring UCLR versus 3% of non-pitchers (*p* < 0.001) [7]. Subsequent surveillance data confirmed persistent upward trends, with 25% of current MLB pitchers and 15% of MiLB pitchers reporting prior UCLR [8].

Incidence is also increasing among adolescent athletes, likely due to year-round play and emphasis on high pitch velocity at younger ages [9]. These trends highlight the need to better define risk factors, refine treatment, and evaluate long-term outcomes [7].

This systematic review and meta-analysis aimed to provide an overview of the incidence of UCL and revision surgeries in baseball players. We examine the current literature to determine the overall rates of these procedures, explore potential risk factors, and identify areas for future research.

## 2. Methods

### 2.1. Eligibility Criteria

This meta-analysis was prospectively registered in the PROSPERO database (ID: CRD420250579421). The methodology conformed to the Preferred Reporting Items for Systematic Reviews and Meta-Analyses (PRISMA) statement [10]. The research objective was structured using the Population, Intervention, Comparison, Outcomes, and Study design (PICOS) model. P (population): Baseball players of any age or level of competition (professional, collegiate, high school, or youth). I (Intervention): Surgical intervention for UCL injury, including primary and revision UCL reconstruction. C (Comparator): No comparator group was required to evaluate the incidence. O (Outcome): Incidence of UCL surgery and incidence of revision UCL surgery. T (time): any study showing incidences in the previous 10 years. S (Study design): Observational studies (cohort, case-control, and cross-sectional).

Specific exclusion criteria were implemented to maintain methodological quality and ensure comparability across the included studies. Individual case reports lacking a cohort-based design were omitted, as were narrative or systematic literature reviews. Multiple publications derived from the same dataset were removed to prevent data duplication. Studies deemed to carry a substantial risk of bias such as those with design flaws, incomplete data reporting, or methodological weaknesses likely to compromise validity were also excluded. Non-shared variables: Studies that did not report relevant variables, such as the incidence of UCL surgery and revision UCL surgery, were excluded. Non-comparable data: Studies with data that could not be compared or pooled with other studies were excluded. Missing data: Studies with incomplete or insufficient data of incidence of UCL surgery, incidence of revision UCL surgery.

### 2.2. Information Sources

A comprehensive literature search was performed across multiple databases, including PubMed, Google Scholar, CINAHL, Embase, and SportDiscus. The search strategy followed a systematic and rigorous approach, without restrictions on publication date or language. In addition, reference lists from the initially selected studies were examined to identify any relevant articles not captured in the primary search.

#### Search Methods for Identification of Studies

The following search terms were applied across all trial registries and databases: (“ulnar collateral ligament” OR UCL) AND pitcher AND (incidence OR prevalence) NOT review NOT “case report” AND surgery (Supplementary File S1). Two independent reviewers (AS, GM) screened the search results and reached consensus on the final selection of eligible studies. The search covered all records from database inception through 20 November 2024.

### 2.3. Selection Process

Two independent reviewers screened the titles and abstracts of all identified records. Full-text articles were retrieved for studies deemed potentially eligible. Inclusion decisions were based on the predefined eligibility criteria, and any disagreements were resolved through discussion and consensus. All included studies reported prior ethical approval or were based on publicly available datasets.

### 2.4. Data Collection Process

Data extraction was independently conducted by two reviewers using a standardized extraction form. Extracted variables included study characteristics (first author, year of publication, study design, and level of evidence), participant characteristics (age, level of play, and playing position), incidence of UCL surgery and revision surgery, identified risk factors, surgical techniques and graft types, return-to-play rates and time to return, and reported complications.

Rothermich et al.’s [11] 2021 study is divided into three seasons: 2016–2017, 2017–2018, and 2018–2019. The 2016–2017 season was already described in Rothermich et al. [12] (2018), while the following seasons were analyzed as separate studies, despite both being in Rothermich et al., 2021 [11]. The 2017–2018 season is represented in the graphs and figures as Rothermich et al., 2021 [11], while the 2018–2019 season was represented in the graphs and figures as Rothermich et al., 2021 [11]. The joint incidences of the three Rothermich et al., 2021 seasons were not evaluated together in case there were significant differences between the seasons.

In Chalmers et al. [13] (2020), we considered the incidence of new cases from pre-2018 to post-2018 and from post-2018 to pre-2019 seasons. We eliminated the 24 that were prior to pre-2018, as there was no indication of when the surgeries were performed.

### 2.5. Data Extraction and Data Items

Two authors independently reviewed the extracted data. Discrepancies were resolved by consulting a third author. Baseline study characteristics collected included study name, region, age at UCLR, years studied, study type, season, position, revision incidence, surgery incidence, and hand. The primary data items of interest were the incidence of UCL surgery and revision UCL surgery. Secondary data included risk factors, surgical techniques, return-to-play rates, and complications.

### 2.6. Risk of Bias in Individual Studies

The methodological quality of the selected studies was evaluated using the Methodological Index for Non-Randomized Studies (MINORS) criteria [Table 1]. This tool assesses essential methodological elements, including study design, patient selection, outcome measurement, and follow-up adequacy. Scores range from 0 to 16 for non-comparative studies and from 0 to 24 for comparative studies. For non-comparative designs, scores of 0–4 were classified as very low quality, 5–7 as low quality, 8–12 as moderate quality, and 13–16 as high quality. For comparative designs, scores of 0–6 indicated very low quality, 7–10 low quality, 11–15 moderate quality, and 16–24 high quality. Disagreements in scoring between the two independent reviewers were resolved through discussion until consensus was achieved.

For randomized controlled trials (RCTs), methodological rigor and risk of bias were independently assessed by two reviewers using the Cochrane Collaboration’s risk of bias assessment tool within Review Manager software (RevMan) [14].

**Table 1:** Assessment of the quality of studies through the Methodological Index for Non-Randomized Studies (MINORS).

Study	Clearly Stated Aim	Consecutive Patients	Prospective Collection Data	Endpoints	Assessment Endpoint	Follow-Up Period	Loss Less than 5%	Study Size	Adequate Control Group	Contemporary Group	Baseline Control	Statistical Analyses	MINORS
Marcus A. Rothermich et al., 2018 [12]	2	2	0	2	2	0	2	2					12
Marcus A. Rothermich et al., 2021 [11]	2	2	0	2	2	0	2	2					12
Mason F. Beaudry et al., 2023 [15]	2	2	0	2	2	0	0	1	2	2	2	2	17
Brandon J. Erickson et al., 2017 [16]	2	2	0	2	2	0	0	1					9
Kevin E. Wilk et al., 2014 [17]	2	2	2	2	2	2	0	1	2	2	2	2	21
Peter N. Chalmers et al., 2020 [13]	2	2	2	2	2	0	0	1					11

### 2.7. Publication Bias Assessment

**Figure 1:**
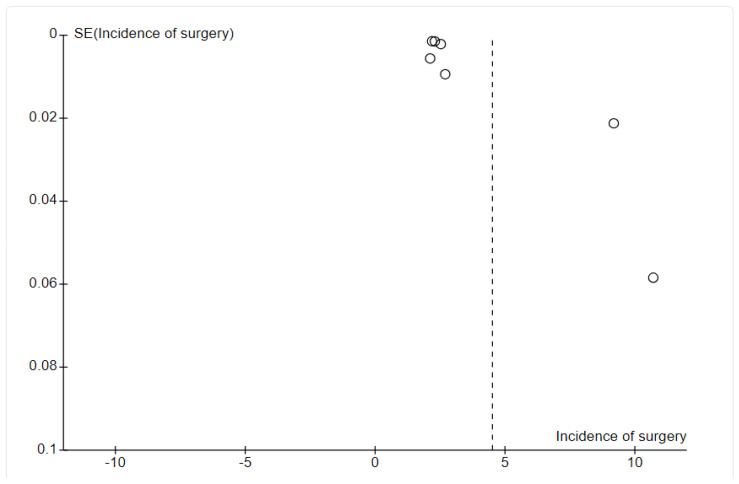
Funnel plot incidence of surgery [11,12,13,15,16,17].

Potential reporting bias was explored through funnel plot analysis performed with Review Manager version 5.4 (Figure 1). Symmetry suggests a low likelihood of substantial publication bias, whereas marked asymmetry could indicate selective non-publication of smaller studies with null or inconclusive findings.

### 2.8. Summary Measures

The following summary measures were used for the meta-analysis:

- Incidence of UCL surgery: Proportion of baseball players undergoing UCL surgery.
- Incidence of revision UCL surgery: proportion of baseball players undergoing revision UCL surgery.

### 2.9. Synthesis of Results

We performed a meta-analysis using RevMan 5.4 software. Pooled proportions and 95% confidence intervals were calculated for the incidence of UCL and revision surgery. Heterogeneity was assessed using the I^2^ statistic.

## 3. Results

### 3.1. Study Selection

**Figure 2:**
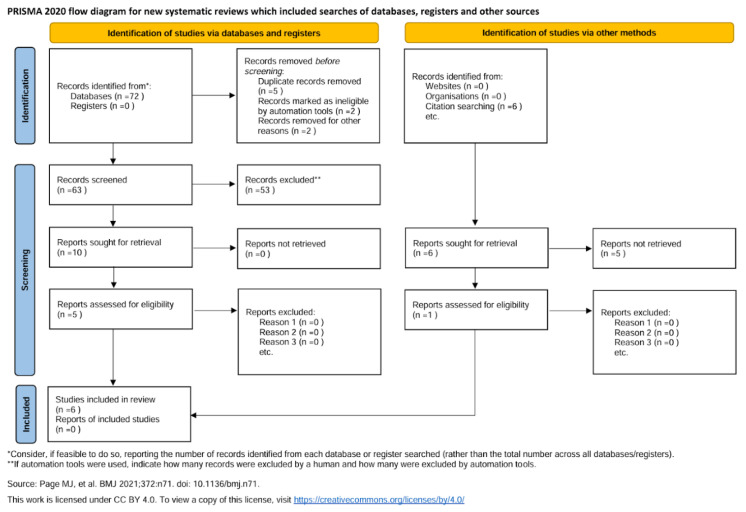
PRISMA flow diagram [10].

The initial search yielded a total of 75 records. After removing duplicates and screening the titles and abstracts, 10 full-text articles were retrieved and assessed for eligibility. Ultimately, six studies met the inclusion criteria and were included in the meta-analysis (Figure 2).

### 3.2. Study Characteristics

The included studies varied in terms of study name, region, age at UCLR, years studied, study type, season, position, revision incidence, surgery incidence, and hand. The primary data items of interest were the incidence of UCL surgery and the incidence of revision UCL surgery. Secondary data items included risk factors, surgical techniques, return-to-play rates, and complications [Table 2].

### 3.3. Additional Analyses

**Figure 3:**
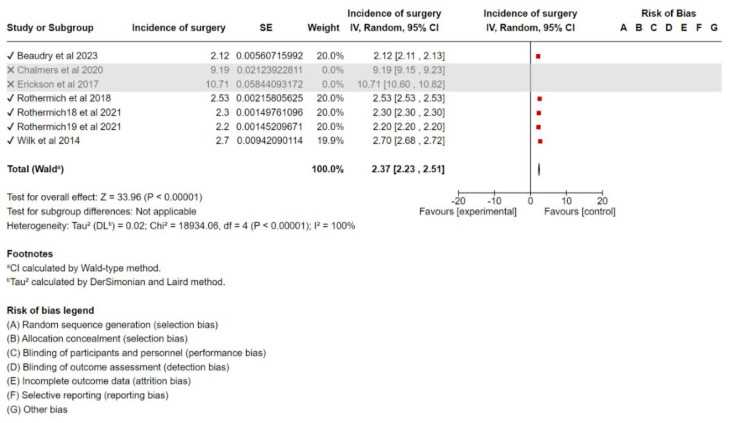
Forest plot of the sensitivity analysis of incidence of UCL surgery [11,12,13,15,16,17].

**Figure 4:**
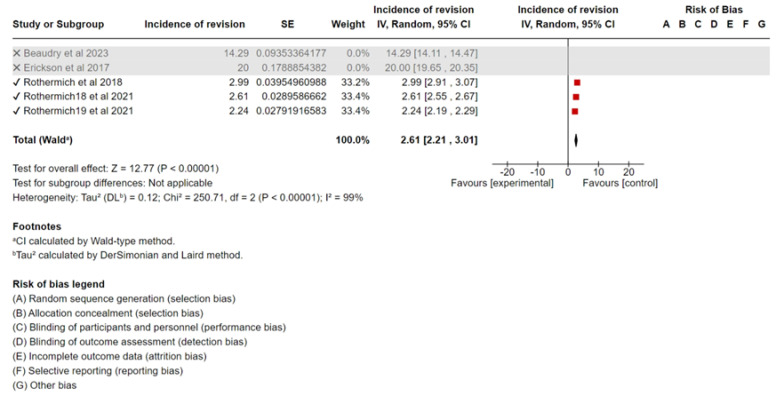
Forest plot of the sensitivity analysis of incidence of UCL surgery [11,12,15,16].

**Figure 5:**
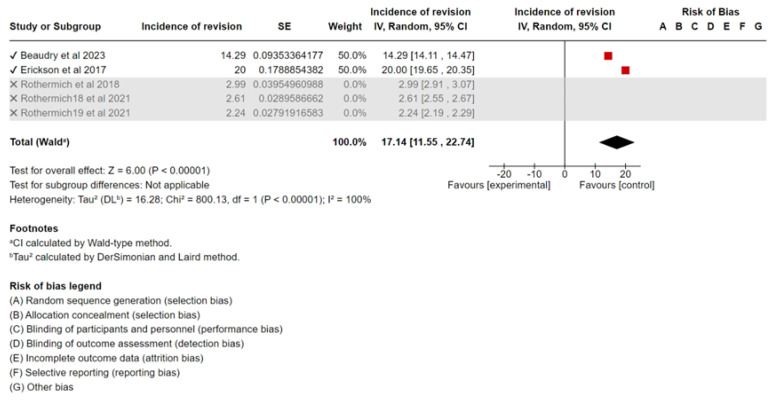
Forest plot of the sensitivity analysis of incidence of revision UCL surgery [11,12,15,16].

Sensitivity analyses were conducted by sequentially excluding the largest contributing trial for each outcome and reanalyzing the data. This procedure, performed in Review Manager 5.4, was intended to assess the robustness of the results and evaluate the impact of excluding individual studies on the overall interpretations (Figure 3, Figure 4 and Figure 5). The methodological quality of the included studies was generally moderate, with some studies having a higher risk of bias due to limitations in the study design or reporting. The MINORS scores of each study are presented in Table 1.

## 4. Results

### 4.1. Study Selection

The initial search yielded a total of 75 articles. After removing duplicates, non-adult population studies, studies unrelated to the incidence of surgery, case reports, and reviews based on titles and abstracts, 62 studies were excluded, resulting in a total of 46 articles. After reviewing the full texts of these 10 articles, 5 studies were excluded for not meeting the inclusion criteria, not sharing variables, having a high risk of bias, and incomplete or incomparable data. Ultimately, five studies were included in the analysis. An additional study was identified on the basis of the references of the included studies. Ultimately, six studies were included in the meta-analysis [Table 1].

### 4.2. Study Characteristics

Table 2 presents the main characteristics of the studies included. Six studies involving 27.366 patients were included. Six of the six studies were published in the USA. Two studies were cohort studies, one was a prospective study, three were descriptive epidemiology studies, and one had a cross-sectional design. The quality of the included studies is listed in Table 1.

### 4.3. Outcomes

For continuous variables measured on the same scale, mean differences (MDs) with corresponding 95% confidence intervals (CIs) were calculated. When measurement scales differed, standardized mean differences (SMDs) were applied. For dichotomous outcomes, odds ratios (ORs) were computed. Statistical heterogeneity across studies was assessed using the I^2^ statistic, with thresholds of 25%, 50%, and 75% representing low, moderate, and high heterogeneity, respectively.

A fixed-effects model was applied when heterogeneity was low (I^2^ < 50%), under the assumption that the included studies estimated a common underlying effect size. When moderate-to-high heterogeneity was present (I^2^ ≥ 50%), a random-effects model was used to account for between-study variability and provide more conservative effect estimates. The choice of model for each pooled analysis was thus guided by the magnitude of heterogeneity and the clinical diversity of the included studies.

Incomplete data reporting across studies was addressed following methodological guidance from the Cochrane Handbook [14].

### 4.4. Publication Bias

Publication bias was assessed using funnel plots, which revealed heterogeneity and publication bias in outcomes, including the incidence of surgery and review (Figure 1). The funnel plot for meta-analysis showed some asymmetry, suggesting potential publication bias.

## 5. Synthesis of Results

### 5.1. Assessment of Results

#### 5.1.1. Incidence of UCL Surgery

**Figure 6:**
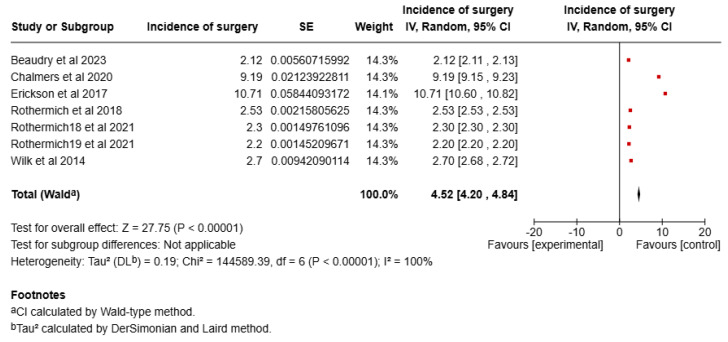
Forest plot of the meta-analysis of incidence of UCL surgery [11,12,13,15,16,17].

The pooled incidence of UCL surgery among baseball players was 4.52% (95% CI: 4.20% to 4.84%). Significant heterogeneity was observed among the studies (I^2^ = 100%, *p* < 0.00001). A forest plot of the meta-analysis is shown in Figure 6.

Sensitivity Analysis: Sensitivity analysis was performed to assess the robustness of the results. After removing studies with the smallest sample size (Erickson et al., 2017 [16], Chalmers 2020 [13]) the meta-analysis resulted in a pooled incidence of 2.37% (95% CI, 2.23–2.51%), which was significantly different from the original analysis. A forest plot of the sensitivity analysis is shown in Figure 3.

#### 5.1.2. Revision Surgery

**Figure 7:**
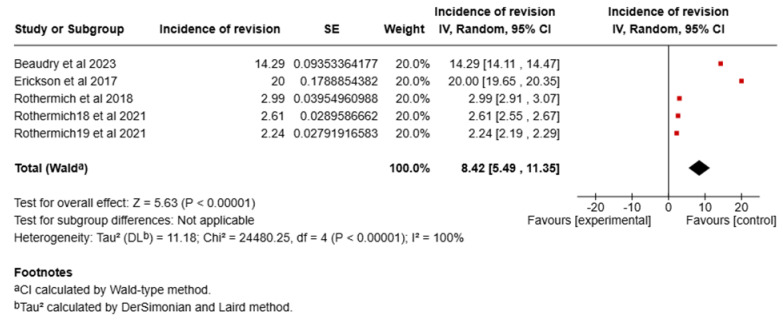
Forest plot of the meta-analysis of incidence of revision UCL surgery [11,12,15,16].

The pooled incidence of revision surgery was 8.42% (95% CI: 5.49% to 11.35%). Significant heterogeneity was observed among the studies (I^2^ = 100%, *p* < 0.00001). A forest plot of the meta-analysis is shown in Figure 7.

#### 5.1.3. Sensitivity Analysis

A sensitivity analysis was performed to assess the robustness of the results. After removing studies with the smallest sample size (Beaudry et al., 2023 [15], Erickson et al., 2017 [16]) the meta-analysis resulted in a pooled incidence of 2.61% (95% CI, 2.21–3.01%), which was not significantly different from the original analysis. A forest plot of the sensitivity analysis is shown in Figure 4. Removing studies with the largest sample sizes (Rothermich et al., 2021 [11], and Rothermich et al., 2018 [12]) from the meta-analysis resulted in a pooled incidence of 17.14% (95% CI, 11.55–22.74%), which was significantly different from the original analysis. A forest plot of the sensitivity analysis is shown in Figure 5.

## 6. Discussion

This meta-analysis provides an overview of the incidence of UCL and revision surgery in baseball players in the current literature. Our findings suggest that UCL surgery is a relatively frequent procedure in baseball players, with a substantial proportion of athletes requiring revision surgery. Although previous research consistently reports rising UCL injury rates, few studies have quantified the actual surgical incidence. Even the success rate of surgery tends to be high; on many occasions it ends the athlete’s career or he/she does not return to the previous level. Therefore, understanding its incidence and types is essential to clarify risk factors.

The rationale for conducting this study is based on determining and understanding the actual incidence of both UCL surgery and revision surgery, which is vital in order to estimate its impact on the world of sports, especially baseball. Understanding the risk and protective factors, taking preventive measures, and increasing the number of studies focused on this topic will help reduce its incidence.

The significant increase in ulnar collateral ligament (UCL) injuries and subsequent reconstructions has emerged as a critical concern in sports medicine, particularly in baseball, where approximately 25% of Major League Baseball (MLB) pitchers have undergone reconstruction (Conte et al., 2015) [7]. This prevalence represents the highest incidence of UCL injuries requiring surgical intervention in all sports (Cain et al., 2010; Fleisig et al., 1995) [18,19], and recent studies have documented a well-established increase across multiple competitive levels, from high school to professional ranks (Ahmad et al., 2003; Azar et al., 2000; Conway et al., 1992; Fleisig et al., 2006; Hechtman et al., 1998; Lyman et al., 2002; Petty et al., 2004; Hodgins et al., 2016) [2,20,21,22,23,24,25,26]. In addition, the number of young baseball players undergoing UCL reconstruction or repair continues to increase. (Wilson et al., 2015; Erickson et al., 2015) [1,3]

As previously mentioned, the most affected players are pitchers (25% MLB; 15% MiLB) (Conte et al., 2015) [7]. Physiological studies have revealed that the UCL in professional pitchers demonstrates dynamic adaptations, becoming thicker and laxer during periods of stress while showing reverse adaptations during rest periods (Wilk et al., 2014) [17]. Recent studies have documented significant increases in both the annual rate of UCL surgery and the prevalence of previous UCL surgery in minor and major league baseball players (Camp et al., 2018; Conte et al., 2016; Leland et al., 2019; Conte et al., 2015; Puga et al., 2024) [6,7,8,27,28]. It should also be noted that the latest studies, such as Puga et al., 2024 [28], mention a decrease in 2023 in the rate of the total number of injuries compared to the 2021 (*p* = 0.01) and 2022 (*p* = 0.02) seasons. There was no statistical difference in the rate of Tommy John surgery, flexor tendon injuries, or elbow injuries due to other causes (Puga et al., 2024) [28].

Several risk factors that would influence the incidence of surgery have been identified, such as warm weather [4]. For example, environmental and physical factors play crucial roles in the UCL injury patterns. Studies have demonstrated that NCAA Division I college baseball pitchers in warm climates undergo UCL reconstruction surgery significantly more frequently and earlier in collegiate careers than those in cold climates (Pescatore et al., 2025) [29]. Additionally, pitch velocity has emerged as the most predictive factor for UCL reconstruction in MLB pitchers, with higher weight and younger age serving as secondary predictors, although these factors collectively explain only 7% of the variance in the reconstruction rates (Zaremski et al., 2015) [30]. Studies have shown that the most common age group to undergo UCLR is between the ages of 15 and 19 years, and this rate has increased to the point of becoming “epidemic” (Ahmad et al., 2003; Cain et al., 2010; Camp et al., 2019; Erickson et al., 2015) [3,6,18,20].

Age-related patterns present particular clinical significance, with studies indicating that younger athletes typically sustain less severe UCL injuries than older players (Zaremski et al., 2017) [31]. The median age at initial diagnosis was 17 years, with a decreasing trend in the median age of patients requiring surgery from 20 to 18 years between 2013 and 2015 (Carr et al., 2022) [32].

Among young players, research indicates that both diversification (playing positions other than the pitcher) and adherence to current pitch-limit regulations may provide protective effects against UCL reconstruction (Beaudry et al., 2023) [15]. However, the progression from Little League World Series pitching to professional baseball remains uncommon, suggesting the need for careful development and monitoring of young players (Beaudry et al., 2023) [15]. For non-professional athletes with closed medial epicondylar growth plates and throwing-related UCL injuries, surgical intervention is more likely, although baseball athletes with partial tears who are skeletally immature require further long-term evaluation (Zaremski et al., 2024) [33].

More players now undergo UCLR after a UCL injury diagnosis (Prodromo et al., 2016) [34]. The annual incidence of UCL reconstruction increased dramatically from year to year (*p* < 0.001), and almost one-third of all procedures performed during the 42-year period were performed from 2011 to 2015 (Conte et al., 2016) [27]. The annual incidence of UCL injuries requiring surgery remains high, with approximately half of collegiate baseball programs involving at least one player undergoing surgery each year (Rothermich et al., 2021) [11]. While most studies report increasing UCL injury rates, few analyze true surgical incidence, which can end careers or prevent return to prior performance level; therefore, it is also necessary to know its incidence and that of the different types of it, to better understand the differences and risk factors that could lead to surgery. The latest research provides us with enlightening and worrying data that should reflect on the incidence of UCL injury and the consequent surgery often necessary for its treatment.

Temporal analysis revealed distinct patterns in UCL reconstruction rates over the past five decades. Three specific change points have been identified in the MLB that occurred during the 1989, 2000, and 2012 seasons (Zaremski et al., 2024) [35]. Similarly, Minor League Baseball demonstrates three change points around 2001, 2009, and 2013, with a notably higher number of UCL reconstructions performed annually in Minor League Baseball compared to MLB (Almonroeder et al., 2024) [36]. Recent data from 2023 suggest a potential decrease in the total number of injuries compared with the 2021 and 2022 seasons, although no statistical difference was observed in Tommy John surgery rates (Puga et al., 2024) [28]. Another study showed seasonal patterns in UCL injuries and demonstrated clear temporal distributions, with most injuries and surgeries occurring during spring. During the baseball season, UCL injury peaked in April/May, followed by a decline, and a second peak in September and October. Surgical interventions show a steady increase from March to June followed by a gradual decrease (Carr et al., 2022) [32]. This seasonal variation suggests potential relationships between injury patterns and playing schedules, which warrants further investigation.

The economic impact of UCL injury is substantial. Analysis of MLB pitchers who underwent reconstruction between 2004 and 2014 revealed an average absence of six months (180.2 days) from the regular season, with total recovery costs reaching USD 395 million and an average of USD 1.9 million per player. Starting pitchers accounted for the largest total cost of recovery at USD 239.6 million, while closing pitchers experienced the highest economic loss per player at USD 3.9 million. Notably, only 77% of pitchers have successfully returned to MLB play (Meldau et al., 2020) [4]. Recent research has emphasized the importance of understanding UCL injury patterns and their implications for treatment decisions. The high frequency of surgical intervention in UCL lesions compared with other common diagnoses [30] underscores the need for refined treatment protocols and prevention strategies. Furthermore, the impact of this pathology extends beyond professional baseball, affecting players at various competitive levels and ages and necessitating a comprehensive approach to injury prevention and management.

## 7. Limitations

This meta-analysis had several limitations. First, the included studies were primarily observational in nature, which limits their ability to draw causal inferences. Second, there was some heterogeneity among the studies in terms of study design and participant characteristics. Third, the available data on the risk factors for UCL injury and revision surgery are limited. As for the main limitations, we can comment on the great lack of studies in which the incidence is studied and reported (our first search shows a reduced number of studies, of which in very few the incidence of surgery is given). Among baseball players, both players and the injury are much more frequent than in PP or other sports, especially in the last 5 years, allowing us to better understand the current incidence and great number of surgeries of UCL of the elbow. However, the six studies included a large number of subjects, thus reflecting a large sample size. More studies are needed to analyze the type of surgery (most of them are UCL reconstruction). Furthermore, the study by Erickson et al. (2017) [16] showed children who did or did not go pro, which we consider to be the period between when they were LLWS players and when they became professional athletes, either in MLB or MiLB. Incidences from Erickson et al. [16] (2017) reflect the incidence of surgery or revision of LLWS in patients who underwent MLB or MiLB. Beaudry et al. [15] 2023 reported the incidence of surgery or revision of only MLB pitchers. In addition, the search also showed that a meta-analysis of the current prevalence could be performed, although the number of studies that discuss prevalence was limited. Only Rothermich’s studies show a more accurate incidence of surgery by season, although the 2018 study shows slight variations compared to the 2021 study, from which the 2017–2018 and 2018–2019 seasons were used as two independent studies (avoiding repeating 2016–2017, already discussed in 2018), which can help us to determine the incidence in professional baseball players (mainly MLB and MiLB). Rothermich et al.’s [11] 2021 study is divided into three seasons: 2016–2017, 2017–2018, and 2018–2019. The 2016–2017 season was already described in Rothermich et al., 2018 [12], while the following seasons were analyzed as separate studies, despite both being in Rothermich et al., 2021 [11]. The 2017–2018 season is represented in the graphs and figures as Rothermich et al., 2021 [11] while the 2018–2019 season was represented in the graphs and figures as Rothermich et al., 2021 [11]. The joint incidences of the three Rothermich et al., 2021 [11] seasons were not evaluated together in case there were significant differences between the seasons. Although the seasons belong to the same research, they are considered and analyzed independently in Rothermich’s study, so data was considered independent and without overlap. In addition, there are hardly any data related to patients undergoing surgery, and only Rothermich’s studies provided more data about the population requiring surgery, which prevented better analysis of other variables that could be related to the incidence of surgery; more studies are needed in different types of baseball players, from those belonging to these leagues, Latin leagues, and outside the USA, especially among adolescents, where the incidence is skyrocketing.

## 8. Conclusions

Despite these limitations, this meta-analysis provides valuable insights into the incidence of UCL and revision surgeries in baseball players. Our findings highlight the need for continued research to better understand the risk factors for these procedures and develop effective prevention strategies. UCL surgery is a relatively common procedure among baseball players, and a substantial proportion of athletes require revision surgery. Our findings highlight the need for continued research to better understand the risk factors for UCL injury and develop effective prevention strategies.

## Figures and Tables

**Table 2:** Baseline characteristics and results of studies on UCL surgery in elbow (incidence UCL surgery).

	Region	Type of Study	Years Studied	Age	USAplayer%	Type Surgery	Season (Moment)	Position	Revision	Incidence of Surgery	Hand
Marcus A. Rothermich et al., 2018 [12]	USA	Descriptive epidemiology study	2016–2017 season	19.7 years. (Age at which surgery was performed)	132/133 (one of them unknown)	Reconstruction: 83.8% (83/99) Repair: 16.2% (16/99) (35/134 total type of surgery unknown)	In-season: 65/134; 48.5% Postseason, offseason, and preseason: 69/134; 51.0%	PP: 19/134; 14.18% Pitchers:(115/134; 85.8%)	4/134	134/5295 (2.54%)	Dominant extremity: (133/134; 99.2%)
Marcus A. Rothermich et al., 2021 [11]	USA	Descriptive epidemiology study	2016–2019	19.9 years (19.7 years in 2017, 20.0 years in 2018, and 19.8 years in 2019).	583/587 USA	UCLR (572/587) players (112/570) UCL repair	In-season 49%-16 to 17 41%-17 to 18 38%-18 to 19; postseason, offseason, and preseason 51%-16 to 17 59%-17 to 18 62%-18 to 19	PP: 19/2757 in 2017, 36/5172 in 2018, and 37/5233 in 2019 Pitchers: 4.4% in 2017 (115/2607), 4.0% in 2018 (194/4847), and 3.7% in 2019 (186/4971).	revision rate in 2017 (4/134), 2.6% in 2018 (6/230), and 2.24% in 2019 (5/223).	(587/25,587)-2.3% 17, 18, 19 2.5% in 2017 (134/5364), 2.3% in 2018 (230/10,019), 2.2% in 2019 (223/10,204)	Dominant extremity (586/587; 99.8%). season 2016 to 2017: 133/134
Mason F. Beaudry et al., 2023 [15]	USA	Cross-sectional study	2019 season	age, 27.39 ± 3.51 years *	USANR	Surgery 11 + 3-Reconstruction 10 + 2 -Other surgeries 1 + 1 other 2/660	NR (not reported)	Pitchers (MLB)	2/660 reinjury (2019)	14/660 (total UCL surgery) -UCLR: 12/660 -Other surgeries: 2/660	Right-handed 311 -TF 177-DD Left-handed 101-TF 71-DD **
Brandon J. Erickson et al., 2017 [16]	USA	Cohort study	2001–2009	11–13 years *	USA 306/638 4.0% [1/25] UCLR Other nationality 332/638 5.4% [2/37] UCLR	NR	NR	Baseball players (Pitcher and PP)	Revision 1/2 pitcher MiLB 0/3MLB	1/3 MBL pitcher 2/25 MiBL pitcher (LLWS that played in MLB) 33%(1/3) required UCLR 8% (2/25) (LLWS that played in MiLB)	NR
Kevin E. Wilk et al., 2014 [17]	USA	Cohort study	2005–2012	Overall 24.7 ± 4.1 Elbow Injury 25.0 ± 4.2 No Elbow Injury 24.7 ± 4.1	NR	NR	NR	NR	NR	8/296 surgery 3/296 UCLR (2 loose-body removals, 2 ulnar nerve transposition, 1 open reduction internal fixation)	NR
Peter N. Chalmers et al., 2020 [13]	USA	Prospective study	pre-2018–post-2018–pre-2019 season	23 years old (pitchers)	NR	NR	NR	Pitchers	NR	17/185 UCLR (pre-2018 to pre-2019). Pre-2018 to post-2018: 10/185 UCLR. Post-2018 to pre-2019: 7/185 UCLR	36/185 left 148/185 right **

* The age of the baseball players included in the study was not the same as that of those operated on. ** Total of players, not the injured/operated. PP: position player (not pitcher); TF: “Tall and Fall”; Pitching Style DD: “Drop and Drive” Pitching Style; LLWS: Little League World Series. NR: not reported.

## Data Availability

All data analyzed in this meta-analysis were extracted from the six included studies (Marcus A. Rothermich et al., 2018 [12]; Mason F. Beaudry et al., 2023 [15]; Brandon J. Erickson et al., 2017 [16]; Kevin E. Wilk et al., 2014 [17]; Peter N. Chalmers et al., 2020 [13]; Marcus A. Rothermich et al., 2021 [11]), which are cited in the manuscript. The pooled incidence rates, methodological quality assessments (MINORS criteria), and study characteristics are presented in Table 1 and Table 2. Raw data from individual studies are publicly accessible in their original publications via PubMed, Embase, and SportDiscus databases. For reproducibility, the RevMan 5.4 analysis files and detailed extraction forms can be requested from the corresponding author (Gonzalo Mariscal) at gonzalo.mariscal@mail.ucv.es.

## Supplementary Materials

The following supporting information can be downloaded at https://www.mdpi.com/article/10.3390/sports13090299/s1: Table S1: PRISMA 2020 Checklist.
